# Acquired Elliptocytosis With Hemolytic Anemia in Myelodysplastic Neoplasm With del(20q)

**DOI:** 10.1111/ijlh.70203

**Published:** 2026-07-25

**Authors:** Nina Sicard, Léa Ousset, Karen Delavigne, Isabelle Luquet, Jean‐Baptiste Rieu

**Affiliations:** ^1^ Centre Hospitalo‐Universitaire (CHU) de Toulouse, Institut Universitaire du Cancer de Toulouse‐Oncopole (IUCT‐O) Université de Toulouse Toulouse France; ^2^ Centre Hospitalo‐Universitaire (CHU) de Toulouse Institut Universitaire du Cancer de Toulouse‐Oncopole (IUCT‐O), Université de Toulouse, UPS, Laboratoire D'hématologie Toulouse France; ^3^ Centre Hospitalo‐Universitaire (CHU) de Toulouse, Institut Universitaire du Cancer de Toulouse‐Oncopole (IUCT‐O) Université de Toulouse, UPS, Service de Médecine Interne Toulouse France

An 83‐year‐old man was referred for pancytopenia (Hb 89 g/L, platelets 76 × 10^9^/L, neutrophils 0.6 × 10^9^/L) associated with laboratory evidence of hemolysis (reticulocyte count 194 × 10^9^/L, haptoglobin < 0.1 g/L, lactate dehydrogenase 356 IU/L, unconjugated bilirubin 22 μmol/L) and marked asthenia. Peripheral blood smear examination A, May–Grünwald–Giemsa, (original magnification ×63) revealed marked poikilocytosis with approximately 30% elliptocytes. No personal or family history of hereditary elliptocytosis was reported and no alternative cause of hemolysis was identified. Bone marrow examination B and C, May–Grünwald–Giemsa, (original magnification ×63) showed erythroid hyperplasia (myeloid: erythroid ratio, 1:2) with marked dyserythropoiesis characterized by nuclear budding, internuclear bridging, multinuclearity, and cytoplasmic vacuolization, together with dysmegakaryopoiesis and dysgranulopoiesis, without excess blasts, consistent with myelodysplastic neoplasm with low blasts and multilineage dysplasia (MDS‐LB‐MLD). Cytogenetic analysis demonstrated a hyperdiploid karyotype (47,XY,+20,ider(20)(q10)del(20)(q11q13)×2), indicating a complex rearrangement of chromosome 20 with duplication of an isoderivative chromosome leading to deletion 20p, partial deletion 20q and partial pentasomy 20q D, (red circle). Next‐generation sequencing identified a *U2AF1* p.Ser34Phe (p.S34F) mutation with a variant allele frequency of 43%. The absence of elliptocytosis on previous blood smears supported the diagnosis of acquired elliptocytosis associated with MDS with del(20q). Treatment consisted of erythropoiesis‐stimulating agents, folate supplementation, and transfusion support as needed. The disease remained clinically stable for nearly 4 years before progression to acute myeloid leukemia (AML).

**FIGURE 1:**
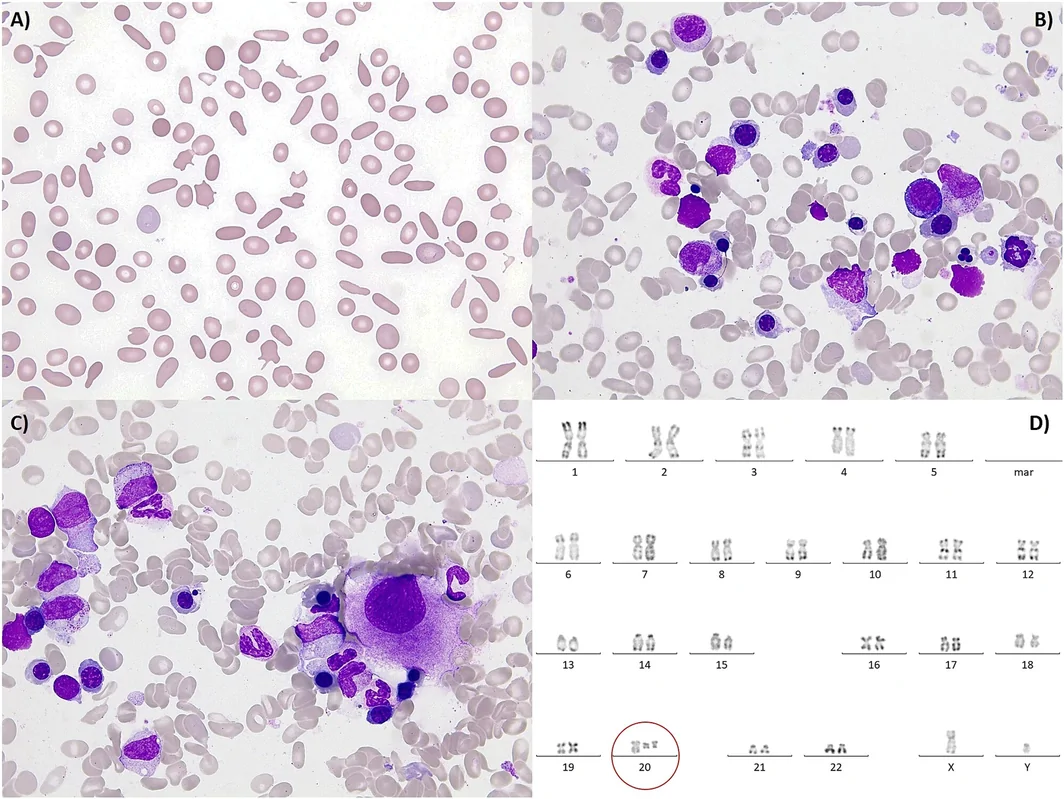
A: Peripheral blood smear examination (May–Grünwald–Giemsa stain, ×63 objective) showing marked poikilocytosis, including approximately 30% elliptocytes. Figures 1. B and C: Bone marrow examination (May–Grünwald–Giemsa stain, ×63 objective) demonstrating dyserythropoiesis with nuclear budding, internuclear bridging, multinuclearity, and cytoplasmic vacuolization; dysmegakaryopoiesis with hypolobated megakaryocytes; and dysgranulopoiesis with hypogranular granulocytes. Figure 1. D: hyperdiploid karyotype (47,XY,+20,ider(20)(q10)del(20)(q11q13)×2), indicating a complex rearrangement of chromosome 20 with duplication of an isoderivative chromosome leading to deletion 20p, partial deletion 20q and partial pentasomy 20q (red circle).

Deletion 20q is a relatively common cytogenetic abnormality in MDS (7%–8% of cases). Although acquired elliptocytosis is rare in MDS, it has repeatedly been reported in association with del(20q). Proposed mechanisms include altered expression of erythrocyte membrane proteins, particularly protein 4.1 (EPB41) [1], and the effects of recurrent mutations such as *U2AF1*, *DNMT3A*, and *TET2* [2]. The cytogenetic breakpoint and extent of chromosome 20 abnormalities may also contribute to the phenotype, potentially explaining why only a minority of MDS with del(20q) develop acquired elliptocytosis. Loss of the chromosome 20 gene *EPB41L1*, which encodes a protein sharing FERM domains with EPB41, has been proposed as a potential contributor in cases involving proximal 20q deletions, although its direct role in erythrocyte membrane integrity remains unproven [3]. While the pathophysiology remains incompletely understood, this case highlights that acquired elliptocytosis in MDS with del(20q) is more than a morphologic curiosity and may be associated with clinically significant hemolysis and symptomatic anemia.

## Author Contributions

N.S. and J.‐B.R. wrote the paper; J.‐B.R. took the pictures; J.‐B.R. and L.O. performed the blood smear and bone marrow examinations; I.L. performed cytogenetic studies, and N.S. and K.D. performed clinical examination and follow‐up of the patient.

## Funding

The authors have nothing to report.

## Disclosure

The authors have nothing to report.

## Ethics Statement

This manuscript respects ethic policy of CHU Toulouse for treatment of human research participants.

## Consent

The authors did not obtain written informed consent from the patient but the patient did not object to his data being used for research purposes (as required by ethic policy of CHU Toulouse).

## Conflicts of Interest

The authors declare no conflicts of interest.

## Data Availability

Data sharing is not applicable to this article as no new data were created or analyzed in this study.

## References

[1] M. Hur , K. M. Lee , H. C. Cho , et al., “Protein 4.1 Deficiency and Deletion of Chromosome 20q Are Associated With Acquired Elliptocytosis in Myelodysplastic Syndrome,” Clinical and Laboratory Haematology 26 (2004): 69–72, 10.1111/j.0141-9854.2003.00583.x. 14738441

[2] C. Robier , G. Hoefler , K. Aubell , and E. Hubmann , “Acquired Elliptocytosis as Presenting Sign of a Myelodysplastic Syndrome Associated With Deletion of Chromosome 20 and Mutations in TET2, DNMT3A, and U2AF1,” Annals of Hematology 100, no. 8 (2021): 2111–2112, 10.1007/s00277-020-04368-w. 33404690

[3] R. Boutault and M. Eveillard , “Acquired Elliptocytosis in the Setting of a Refractory Anemia With Excess Blasts and del(20q),” Blood 127, no. 21 (2016): 2646, 10.1182/blood-2016-03-703876. 28092889

